# A Multi-Isotopic Chemometric Approach for Tracing Hazelnut Origins

**DOI:** 10.3390/foods13213399

**Published:** 2024-10-25

**Authors:** Berta Torres-Cobos, Mònica Rosell, Albert Soler, Mercè Rovira, Agustí Romero, Francesc Guardiola, Stefania Vichi, Alba Tres

**Affiliations:** 1 Departament de Nutrició, Ciències de l’Alimentació i Gastronomia, Universitat de Barcelona, Av Prat de La Riba, 171, 08921 Santa Coloma de Gramenet, Spain; bertatorres@ub.edu (B.T.-C.); fguardiola@ub.edu (F.G.); atres@ub.edu (A.T.); 2 Institut de Recerca en Nutrició i Seguretat Alimentària (INSA-UB), Universitat de Barcelona, Av Prat de La Riba, 171, 08921 Santa Coloma de Gramenet, Spain; 3 Grup MAiMA, Mineralogia Aplicada, Geoquímica i Hidrogeologia, Departament de Mineralogia, Petrologia i Geologia Aplicada, Institut de Recerca de l’Aigua (IdRA), Universitat de Barcelona, Martí i Franqués s/n, 08028 Barcelona, Spain; monica.rosell@ub.edu (M.R.); albertsolergil@ub.edu (A.S.); 4 Institute of Agrifood Research and Technology (IRTA), Ctra. de Reus–El Morell Km 3.8, 43120 Constantí, Spain; merce.rovira58@gmail.com (M.R.); agusti.romero@irta.cat (A.R.)

**Keywords:** hazelnut, stable isotopes, geographical authentication, PLS-DA, food fraud

## Abstract

High-value products, such as hazelnuts, are particularly vulnerable to fraud due to their price dependence on geographical origin. Guaranteeing hazelnuts’ authenticity is essential for consumer trust and safety. Stable isotope analysis has become a reference method for origin authentication as it is reliable, robust, and easily transferable across laboratories. However, multiple isotopic markers coupled with chemometric techniques are often needed to authenticate food provenance accurately. In this study, we focused on assessing the potential of bulk δ^18^O, along with δ^2^H and δ^13^C of the main fatty acids, as hazelnut-origin authenticity markers. PLS-DA classification models were developed to differentiate samples (n = 207) according to their region of origin. This multi-isotopic approach provided promising external validation results, achieving a 94% global correct classification rate in discriminating hazelnuts from regions with distinct geographical and environmental conditions. This study lays the groundwork for further model development and evaluation across additional production areas and harvest years.

## 1. Introduction

Ensuring the authenticity of food products is paramount for fostering consumer trust and safety throughout the food chain. Verifying authenticity and traceability demands robust analytical methods, which is especially crucial for regulatory bodies. Over the past few decades, stable isotope analysis has progressively consolidated its position as a reference authentication method, as isotopic data can be reliably measured in routine work and effectively compared across laboratories. This reliability has also led to the utilization of these methods in legal cases as part of enforcement exercises [1]. According to various studies, stable isotope analysis is effective at detecting adulteration and verifying farming practices in various food products [2,3,4,5], as well as in determining the geographical origin of food commodities [1,6,7,8,9,10,11], which is a crucial aspect of assessing food authenticity. The effectiveness of isotopic measurements in verifying food’s geographical origin stems from the dependence of isotopic ratios on the geological characteristics and environmental conditions of the area where it was produced. Regarding plant-derived food specifically, the isotopic ratio of light elements (δ^13^C, δ^2^H, and δ^18^O) is significantly shaped by geological and environmental conditions, such as soil composition and precipitation patterns [4,6,7,8,9,10,12]. The δ^13^C ratio in plants is closely related to their botanical origin, allowing discrimination between C3 and C4 plants, with the former being more depleted in the heavier isotope [13,14]. However, other studies have shown that it might be influenced by several environmental factors such as temperature, precipitation, water stress, humidity, and ambient CO_2_ concentration [15,16,17]. The δ^2^H and δ^18^O values in plants are determined by the meteoric water isotopic ratios of the specific cultivation territory. These ratios result from isotopic fractionation processes occurring during water phase changes (solid–liquid–vapor) such as evaporation, condensation, and precipitation, which are driven by factors such as latitude, altitude, and temperature [6,8]. Nevertheless, while organic hydrogen (H) in plants derives solely from water in the hydrosphere, oxygen (O) can originate from other sources, such as the atmosphere or photosynthesized CO_2_ [8,10]. Therefore, numerous environmental and climatic factors can impact the δ^18^O ratio, rendering it a particularly promising candidate for geographical authentication. 

Recent studies have shifted their focus beyond the bulk analysis of light bio-elements to include compound-specific isotopic data [4,10]. This approach allows for more insight into isotopic data by assessing the isotopic ratios of elements in specific food components such as proteins, lipids, or carbohydrates [4]. This strategy has been applied for origin authentication in a wide number of foods, including honey [18], olive oil [10,19], pumpkin [20], and shitake mushrooms [21], among others. 

Geographical origin determination often requires considering multiple stable isotope parameters [1]. In fact, in most cases, a single isotopic marker is insufficient to authenticate food origin [7,9,10,22], but the combination of multiple isotopic markers can satisfactorily achieve this purpose, offering more comprehensive information and enhancing method accuracy.

In addition to selecting the appropriate isotopic markers, a crucial aspect of the authentication strategy lies in data analysis, especially when dealing with multi-isotope ratios. Supervised chemometric techniques, such as partial least squares discriminant analysis (PLS-DA), offer an advantageous alternative to unsupervised methods, enabling the construction of classification models and the accurate prediction of unknown samples. Specifically, PLS-DA is focused on finding the maximum correlation between data and the selected class variable, minimizing those variables not related to the selected category. It has been proven to be an efficient tool for authentication purposes [23,24,25]. Various studies have successfully applied multi-isotope analysis combined with chemometrics to verify the origin of various food products, aiming to protect consumers from misleading information [6,7,8,9,11,23,24,25]. 

Given that market prices frequently rely on the geographical area of production, certain high-value products become particularly vulnerable to fraudulent practices, such as falsifying a label’s declared origin. This is motivated by the potential for illicit profit and is often aggravated by a lack of efficient authentication methods. This is the case with hazelnuts, whose price and sensorial characteristics are highly dependent on their region of origin [26,27]. These nuts are highly valued in the food industry, both in their raw and processed forms. Italian and Spanish hazelnuts, in particular, present market prices up to twice as high as those from other origins [27], with hazelnuts under quality schemes, such as Protected Denominations of Origin and Protected Geographical Indications, further enhancing their value [28]. 

Few studies have addressed nut authenticity through isotope-based methods. Promising findings have been obtained from analyzing the isotopic ratios of bio-elements in pistachios [29], walnuts [12,30,31], peanuts [32], and pine nuts [33]. To the best of the authors’ knowledge, only Sammarco et al. [34] have applied isotopic markers to authenticate the origin of hazelnuts, combining this approach with multi-elemental analysis. To further explore the potential of isotopic data in verifying hazelnuts’ origin, preliminary research was carried out to optimize the analytical procedures and to identify the most suitable isotopic markers for implementation in a multi-isotopic approach [35]. This prospective study, conducted on a restricted sample set, revealed that bulk δ^18^O, together with δ^2^H and δ^13^C measured in the main fatty acid methyl esters (FAMEs) were the most relevant in discriminating hazelnut samples from different origins when excluding isotopic markers influenced by external factors such as fertilization treatment. Therefore, exploring the potential of multi-isotopic markers by constructing and testing origin classification models based on large sample sets, including different provenances and harvest years, could provide new, reliable tools to ensure hazelnut origin.

Considering this previous knowledge, the main aim of the present study is to explore the potential of several isotopic markers that have formerly shown promise as hazelnut geographical authentication tools and to assess their suitability. For this purpose, PLS-DA classification models based on bulk δ^18^O, along with δ^2^H, and δ^13^C of the main FAMEs were developed to discriminate hazelnuts according to their region of origin. These models were built on a sample set of 207 hazelnuts from four different geographical origins and evaluated through external validation. 

## 2. Materials and Methods

### 2.1. Samples

The sample set consisted of 207 ‘Tonda di Giffoni’ hazelnuts from four harvest seasons and four different regions located in Chile (CHL, n = 40; 2019, 2020), Spain (ESP, n = 91; 2019, 2020, 2021, 2022), Georgia (GEO, n = 40; 2021, 2022), and Italy (ITA, n = 36; 2019, 2020, 2021), respectively. Geographical coordinates for each production region are reported in Table 1. 

Each hazelnut sample was taken from an individual tree and collected at the time of ripening, with harvests taking place in March–April for CHL and in August–October for ESP, GEO, and ITA. Sample information is summarized in Table S1 of the Supplementary Materials.

Samples were obtained within the TRACENUTS project (PID2020-117701RB-I00) and collected from distinct hazelnut trees by producers. Hazelnuts were shelled at the laboratory, and kernels were stored under vacuum at 4 °C until analysis.

### 2.2. Bulk Isotopic Analysis by Elemental Analysis–Isotope Ratio Mass Spectrometry (EA-IRMS)

#### 2.2.1. Sample Preparation for Bulk Isotopic Analysis

Sample preparation was carried out, as reported by Torres-Cobos et al. [35]. About 30 g of raw hazelnuts were ground into fine powder using a domestic grinder (Aromatic, Taurus, Oliana, Spain). Then, ground samples were lyophilized for three days in a Telstar Cryodos-45 freeze dryer (Telstar, Terrassa, Spain). 

#### 2.2.2. EA-IRMS 

Samples were analyzed in the conditions reported by Torres-Cobos et al. [35]. An aliquot of 0.3 mg of the ground sample was placed into silver capsules (Lüdi Swiss, Flawil, Switzerland) and analyzed in a TC/EA-IRMS Delta Plus XP (Thermo Fisher Scientific) equipped with an autosampler (Sample Tray, N° 2, MAS200R autosampler) and a 450 mm ceramic pyrolysis reactor, heated to 1445 °C. The filling of the reactor was carried out according to the Thermo Fisher Scientific manual [35]. Helium was the carrier gas (pressure 62 kPa), and carbon monoxide (CO) was the reference gas with a δ^18^O-value of −8.68 ‰ (pressure 180 kPa). The certified standard IAEA-601 (benzoic acid, δ^18^O = +23.3 ‰) and the internal secondary standards, UB-YCEM (δ^18^O = +17.6 ‰) and UB-ASC (δ^18^O = +13.2 ‰), both barium sulfates, were used fitting the range of samples. Samples were analyzed in duplicate.

### 2.3. Isotopic Analysis of FAMEs by Gas Chromatography–Isotope Ratio Mass Spectrometry (GC-IRMS)

#### 2.3.1. Sample Preparation for Isotopic Analysis of FAMEs

Samples were prepared, as reported by Torres-Cobos et al. [35]. For each sample, the lipid fraction of 25 g of ground hazelnuts was extracted with 50 mL of diethyl ether and evaporated to dryness. Subsequently, FAMEs were prepared, dissolving 100 mg of hazelnut oil in 2 mL of hexane and adding 200 μL of a 2M methanolic potassium hydroxide solution [2]. After centrifugation, the supernatant was analyzed.

#### 2.3.2. GC-IRMS

The analysis was carried out as reported by Torres-Cobos et al. [35]. The H and C isotopes of individual FAMEs were analyzed in duplicate using a Trace GC Ultra gas chromatograph with a Triplus Autosampler, coupled to an Isotope Ratio Mass Spectrometer Delta V Advantage through a GC Isolink interface (Thermo Fisher Scientific, Waltham, MA, USA). The injection volume was 1 μL, with a split ratio of 1:5. The separation of FAMEs was performed on a VF-23ms capillary column (60 × 0.32 mm I.D., 0.15 μm of Agilent Technologies, Santa Clara, CA, USA). The GC oven temperature was initially set at 60 °C, held for 1 min, increased to 160 °C at a rate of 6 °C/min, and held for 10 min. Lastly, it was raised to 240 °C at a rate of 6 °C/min. 

Helium was the carrier gas at a flow rate of 1.8 mL/min. The injector temperature was 240 °C. The commercial NiO/CuO-NiO-Pt combustion reactor (P/N 1255321, Thermo Fisher Scientific, Waltham, MA, USA) operated at 1000 °C for CO_2_. For the analysis of H_2_, the commercial high-temperature reactor (P/N 1255330, Thermo Fisher Scientific, Waltham, MA, USA) was used and adjusted to 1400 °C. The certificated standards used for the analysis of δ^2^H were Androstane (δ^2^H = −293.2 ± 1.0 ‰), USGS76 (δ^2^H = −210.8 ± 0.9 ‰), Coumarin (δ^2^H = 82.3 ± 1.2 ‰), and a secondary standard, FAME C19 (δ^2^H = −215.25 ± 0.6 ‰). For the analysis of δ^13^C, the standards used were Icosane (δ^13^C = −40.91 ± 0.02 ‰), FAME C16 (δ^13^C = −30.78 ± 0.02 ‰), USGS76 (δ^13^C = −31.36 ± 0.04 ‰), FAME C19 (δ^13^C = −30.32 ± 0.02 ‰), USGS72 (δ^13^C = −1.54 ± 0.03 ‰), and Phenanthrene 16/0020 (δ^13^C = −23.90 ± 0.14 ‰).

### 2.4. Data Treatment and Statistical Analysis

A data matrix was built, with samples in the rows (n = 207) and variables in the columns (bulk δ^18^O; δ^13^C of C16:0, C18:0, C18:1 and C18:2 FAMEs; and δ^2^H of C16:0, C18:1 and C18:2 FAMEs). To study the effect of interannual variability and the differences in isotopic ratios across origins, independent sample t-tests and one-way analysis of variance (ANOVA), together with Scheffé’s post hoc test, were performed in IBM SPSS Statistics v29.0© (IBM Corp., Armonk, NY, USA) for isotopic ratios that followed a normal distribution according to the Shapiro–Wilk normality test, whereas the Mann–Whitney U and Kruskal–Wallis tests were applied to those variables that did not present a normal distribution to determine if there were significant differences between the isotopic ratios throughout the different harvest years and origins.

Model development was performed on SIMCA v13.0© (Sartorius, Göttingen, Germany). Principal Component Analysis (PCA) was used for preliminary data exploration and to detect possible outliers based on Hotelling’s T^2^ range and model residual parameters. 

The sample set was randomly split into the training set (80% of the samples of each class, n = 166) and validation set (20% of the samples of each class, n = 41), assuring that samples from all origins and harvest years were included in both sets. This splitting was run seven times (seven iterations) to evaluate the effect of the sample’s set composition and to increase the robustness of external validation. The sample set splitting information, validation, and training sets are summarized in Table S1 of the Supplementary Materials.

Partial least square–discriminant analysis (PLS-DA) classification models were developed and validated to classify samples according to their geographical origin (SIMCA v13.0©). First, a PLS-DA model was built with each training set (n = 166) to discriminate between the four origins: Chile (CHL, n = 32), Spain (ESP, n = 73), Georgia (GEO, n = 32), and Italy (ITA, n = 29), respectively (see Section 3.1). After evaluating the results from this four-class model, a three-class model was developed to discriminate between CHL, GEO, and a third class that combined both ESP and ITA samples.

In PLS-DA multi-class models, a dummy Y matrix is employed, featuring classification vectors equivalent to the number of classes. Each vector assigns a value of 1 to a specific class (representing a specific region of origin) and 0 to all other classes (representing other regions). Afterward, each sample is categorized into the class associated with the vector yielding the highest PLS predicted value (PV), provided it is higher than the classification threshold. Samples failing to surpass the classification threshold for any vector remain unassigned to any region (no class).

To optimize the classification thresholds and maximize the classification performance, receiver operating characteristics (ROC) analysis was applied to PLS-DA models to set the thresholds of each class. Hence, ROC curves (one for each training model and each class) were built up with the PV obtained in the leave 10% out cross-validation and the real class for each sample in each model. The ROC curve plots the sensitivity and 1-specificity values obtained when the PV threshold that assigns samples to a class varies. Then, the selected threshold for each class and each model corresponds to the one that maximizes the sensitivity and specificity of the classification [36]. 

Models developed with the training sets were internally validated by the leave 10% out cross-validation method. The optimal number of latent variables and pre-processing were selected according to the lowest Root Mean Squared Error of Cross-Validation (RMSEcv) criteria. Model overfitting was assessed through the permutation test (n = 20 permutations) and the ANOVA of the cross-validated predictive residuals (*p*-value). No outliers were detected according to Hotteling’s T^2^ range and model residual parameters. For all models, the optimal pre-processing, according to the lowest RMSEcv, was mean-centering and scaling to the unit of variance. The permutation test and ANOVA *p*-value results showed that none of the training models were overfitted.

Finally, the external validation was carried out using each training model to predict the class of samples in the complementary validation set. The performance of the PLS-DA models was evaluated by the Q^2^ values and efficiency in external validation, which was expressed as the percentage of correct classification in each class.

## 3. Results and Discussion

With the aim of developing an efficient multi-isotopic tool for the geographical authentication of hazelnuts, the isotopic markers that demonstrated the highest discriminatory power were selected in the previous prospective study [35], which include bulk δ^18^O, as well as δ^2^H and δ^13^C of the main hazelnut FAMEs. By focusing exclusively on the most discriminating markers, this approach streamlines workload optimization and facilitates the method’s application in routine controls. To gain insight into the behavior of the different isotopic markers prior to constructing the multi-isotopic model, Table 2 presents their mean and standard deviation results across the four origins and the significant differences identified with the corresponding statistical tests (ANOVA and Kruskal–Wallis). Noteworthy, differences between means according to the origin, particularly for bulk δ^18^O and δ^2^H of the FAMEs, highlight the discriminatory potential of these markers. However, although the means were significantly different between two or more countries, no individual markers could independently distinguish between all origins. This reinforces the validity of our approach, which relies on a multi-isotopic analysis combined with multivariate techniques to comprehensively assess the origin of hazelnuts. In addition, significant differences were found between the means of the isotopic ratios of different years for the same geographical region (Table S2 of the Supplementary Materials), confirming the need to include multiple harvest years in the model building in order to consider the interannual variability and increase the robustness of the models in the prediction of future samples. 

**Figure 1:**
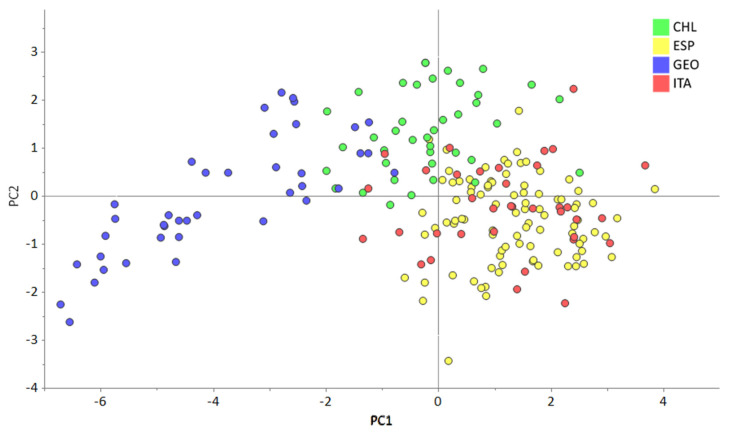
Score plot of the first and second principal components of the PCA model developed using bulk δ^18^O data and δ^2^H, δ^13^C data of the main fatty acid methyl esters.

An examination of the score plot of the exploratory PCA (Figure 1) revealed a clear separation between the GEO and CHL clusters, while ESP and ITA samples formed an overlapping cluster. This initial exploration suggested that the isotopic profiles of the hazelnuts from regions in Spain and Italy displayed minimal differences and were unlikely to be easily distinguishable by the PLS-DA classification model. Nevertheless, a PLS-DA to discriminate between the four origins was attempted. 

### 3.1. Four-Class PLS-DA Model

The internal validation results obtained from seven iterations revealed that the four-class PLS-DA model was unable to effectively discriminate among all the tested origins, with correct classification rates of 66.1%, 86.9%, 100%, and 19.7% for CHL, ESP, GEO, and ITA samples, respectively (Table S3 of the Supplementary Materials). In particular, the model failed at classifying ITA hazelnuts, predominantly misclassifying them as ESP samples or failing to assign them to any class. This outcome could be due to the similarities between the Spanish and the Italian regions studied. Indeed, both regions share similar climatic and environmental conditions, including proximity to the coast, low altitude, and Mediterranean climate. These similarities are further evidenced by the isotopic ratios of the precipitations in these particular areas: ITA (δ^2^H_water_ = −38‰, δ^18^O_water_ = −6.2‰) and ESP (δ^2^H_water_ = −34‰, δ^18^O_water_ = −5.6‰). However, the values in the GEO regions (δ^2^H_water_ = −53‰, δ^18^O_water_ = −8.4‰) and CHL (δ^2^H_water_ = −41‰, δ^18^O_water_ = −6.4‰) differ considerably [37,38,39]. The above-mentioned analogies were reflected in the ITA and ESP isotopic profiles, making them indistinguishable from the PLS-DA model. Although stable isotope analysis is a useful tool to discriminate samples from different geographical regions, it becomes challenging when these regions are closely situated and share similar pedoclimatic conditions, latitude, altitude, or proximity to the sea. Despite the fact that distinguishing between similar regions may be challenging, it is valuable to differentiate them from other regions with distinct geographical and environmental conditions. Specifically, counterfeiting Spanish and Italian hazelnuts among themselves, given their similar market prices, would not be as profitable as counterfeiting them with significantly cheaper hazelnuts, such as those from Chile and especially Georgia. Thus, it remains crucial to distinguish between highly priced hazelnuts (ESP and ITA) and hazelnuts from these other regions. For this reason, we merged ESP and ITA classes into one class (ESP + ITA) and built PLS-DA models with three classes to classify samples into GEO, CHL, and ESP + ITA. The optimal thresholds for each class are summarized in Table S4 of the Supplementary Materials. Alternatively, if distinguishing the Spanish region from the Italian region was specifically required, other stable isotopes with strong geogenic connections, such as Strontium (Sr), could additionally be considered according to the promising results from our previous study [35].

### 3.2. Three-Class PLS-DA Model

**Figure 2:**
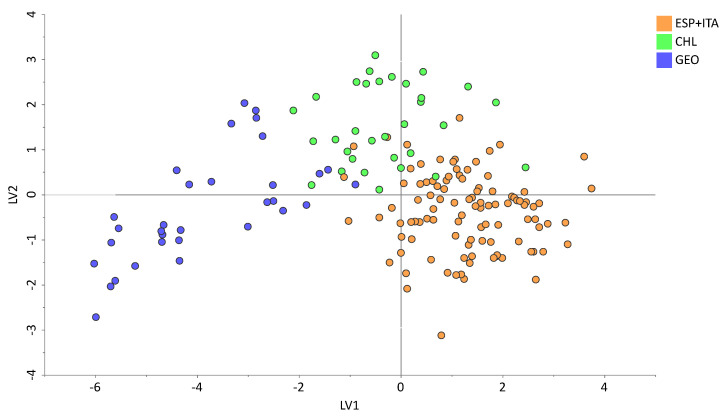
Score plot of the first and second latent variables of the PLS-DA model developed using bulk δ^18^O data and δ^2^H, δ^13^C data of the main fatty acid methyl esters.

The mean results of the leave 10% out cross-validation of the seven training sets are reported in Table S5 of the Supplementary Materials. The internal validation presented promising outcomes, achieving a 95.9% global correct classification and percentages equal to or higher than 87.5% for all the individual classes. Figure 2 displays the score plot of the PLS-DA model. However, the proper validation of authentication models requires external validation, assessed through the prediction of samples that have not been used to build the models, such as a validation set.

External validation results, expressed as the mean and standard deviation obtained from the seven iteration sets, are summarized in Table 3. The model was successful at classifying the samples into three classes: CHL, GEO, and ESP + ITA. A 93.7% global correct classification rate was achieved with a low standard deviation, indicating the good discriminant capacity of the model, with minimal dependency on the composition of the validation set. No annual effects were observed in the misclassified samples as there were wrongly classified samples from all years (Table S6 of Supplementary Materials) despite the interannual differences observed for some isotopic markers and origins (Table S2 of Supplementary Materials).

The model was especially successful in classifying GEO samples (100%), reflecting the significant differences in all isotopic ratios compared to other origins (Table 2), particularly for lower bulk δ^18^O values, consistent with the δ^18^O precipitation values across the studied areas (GEO δ^18^O_water_ = −8.4‰; CHL δ^18^O_water_ = −6.4‰; ITA δ^18^O_water_ = −6.2‰; ESP δ^18^O_water_ = −5.6‰) [37,38,39]. However, while none of these differences alone were sufficient to distinguish GEO from other origins, the combination of all isotopic markers into a PLS-DA model enabled a complete differentiation, further ratifying the hypothesis that a multi-isotopic chemometric approach is crucial for geographic classification. 

Moreover, the model successfully classified 82.1% of the CHL samples despite the similar δ^18^O and δ^13^C isotopic ratios of the main FAMEs in the ESP + ITA class, leading to the occasional misclassification of CHL hazelnuts into this class. The successful discrimination of CHL and ESP + ITA samples may be primarily attributed to the notable differences observed in the mean δ^2^H values of the FAMEs as all three isotopic ratios (δ^2^H_Palmitic_, δ^2^H_Oleic_ and δ^2^H_Linoleic_) showed significant differences between CHL and ESP + ITA (Table 2). Specifically, and considering associated errors normally <6‰, the δ^2^H_Oleic_ values were more strongly enriched (around −175 and −180‰) for the ESP + ITA class than for the CHL class (around −190‰). The same was true for δ^2^H_Palmitic_ and δ^2^H_Linoleic_ values, approximately −165‰ and −200 ‰ for the ESP + ITA, and −179‰ and −215‰ for the CHL, respectively. These findings align with the δ^2^H precipitation values registered in each region, which showed slight variations. CHL exhibited the most depleted δ^2^H values (δ^2^H_water_ = −41‰) compared to ITA (δ^2^H_water_ = −38‰) and ESP (δ^2^H_water_ = −34‰) [37,38,39]. However, besides precipitation, other factors can influence the δ^18^O and δ^2^H of FAME isotopic ratios. The δ^18^O values of the samples are not only derived from water in the hydrosphere but can originate from multiple sources, such as photosynthesized CO_2_ or atmospheric O_2_. Additionally, although the only source of organic hydrogen for plants comes from water in the hydrosphere, this hydrogen is metabolized by different synthetic routes to synthesize fatty acids, resulting in different ratios for each fatty acid [8,40,41]. 

Although the isotopic ratios of bio-elements might be influenced by many factors, these results show their potential as a tool for verifying the geographical origin of hazelnuts across regions with diverse geological and environmental conditions.

## 4. Conclusions

In this study, we evaluated the suitability of a multi-isotopic approach combined with chemometrics as a tool for authenticating hazelnut geographical origin. This assessment involved the analysis of bulk and compound-specific isotopic markers (bulk δ^18^O, together with δ^13^C and δ^2^H of the FAMEs) using a set of 207 samples from regions located in four different countries and across different harvest seasons. 

The results showed that the differences in δ^18^O and δ^2^H of the FAMEs seemed coherent with the precipitation data of the four territories, which proved to be useful for discriminating hazelnuts according to origin. When the considered regions were sufficiently distinct in terms of climate and geography, this approach proved valuable for distinguishing hazelnuts originating from various sources. This was demonstrated by the promising results obtained in classifying regions located in the Mediterranean basin (ESP + ITA) from those in CHL and GEO. These regions could be accurately classified with a 93.7 ± 4.2% of the global correct classification rate, proving the method to be reliable and not set-dependent. 

On the other hand, it is important to note that regions with similar pedoclimatic characteristics may present challenges in distinguishing them based on their isotopic ratios of light elements. This was evident in the regions studied in this research, situated in Spain and Italy, where the differences in analyzed isotopic ratios were not significant enough for differentiation. 

These findings suggest that, provided the regions are sufficiently distinct in climate and geography, the further development of a reliable and universally applicable method for identifying the geographical origin of hazelnuts would be feasible and could effectively detect counterfeit products from different sources. Nevertheless, this shall require further evaluation of the model by including samples from additional production areas. Also, as the isotopic ratios depend on the climatic conditions, it would be advisable to regularly update the models by including future harvest years. This step is essential to implement the model as a reliable authentication tool to support inspections verifying the geographical origin of hazelnuts.

## Figures and Tables

**Table 1:** Harvest year and geographical coordinates of parcels where hazelnuts were produced.

	2019 (n)	2020 (n)	2021 (n)	2022 (n)	Geographical Coordinates
Chile	20	20	-	-	35°15′36″ S, 71°32′60″ W
Spain	23	23	23	22	41°10′15″ N, 1°10′09″ E
Georgia	-	-	20	20	42°27′34″ N, 41°51′31″ E
Italy	12	12	12	-	42°25′23″ N, 12°4′45″ E

**Table 2:** Mean and standard deviation results and differences in isotopic ratios across the various origins.

Isotopic Signatures	Chile (n = 40)	Spain (n = 91)	Georgia (n = 40)	Italy (n = 36)
δ^18^O (‰)	23.7 ± 0.7 ^b^	23.6 ± 0.8 ^b^	19.8 ± 0.5 ^c^	24.8 ± 0.8 ^a^
δ^13^C_Palmitic_ (‰)	−29.9 ± 0.7 ^a^	−29.9 ± 0.5 ^a^	−31.8 ± 1.2 ^b^	−29.9 ± 0.6 ^a^
δ^13^C_Stearic_ (‰)	−31.0 ± 1.2 ^ab^	−30.8 ± 1.1 ^a^	−32.8 ± 1.1 ^c^	−31.3 ± 1.2 ^b^
δ^13^C_Oleic_ (‰)	−28.4 ± 0.6 ^a^	−28.4 ± 0.5 ^a^	−30.3 ± 1.3 ^b^	−28.3 ± 0.6 ^a^
δ^13^C_Linoleic_ (‰)	−30.5 ± 0.7 ^a^	−30.5 ± 0.7 ^a^	−32.7 ± 1.6 ^b^	−30.5 ± 0.9 ^a^
δ^2^H_Palmitic_ (‰)	−179.0 ± 6.8 ^b^	−163.0 ± 8.2 ^a^	−186.6 ± 6.9 ^c^	−166.5 ± 8.2 ^a^
δ^2^H_Oleic_ (‰)	−191.3 ± 5.3 ^c^	−174.8 ± 5.4 ^a^	−197.5 ± 5.3 ^d^	−179.4 ± 6.9 ^b^
δ^2^H_Linoleic_ (‰)	−215.3 ± 5.8 ^b^	−199.5 ± 6.7 ^a^	−219.4 ± 5.0 ^c^	−200.7 ± 7.7 ^a^

In all cases, *p*-values were <0.05 (according to ANOVA or Kruskal–Wallis). Significant differences between origins were noted with different superscript letters in the same row as a > b > c > d.

**Table 3:** Results of the external validation of three-class PLS-DA models. Results are mean values (± standard deviation) obtained from seven iterations.

True Origins		Origins Assigned by the Model				
	n	CHL (n)	ESP + ITA (n)	GEO (n)	Not Assigned (n)	Correct Classification (%)
CHL	8	6.6 ± 1.1	1.3 ± 1.0	0.0 ± 0.0	0.1 ± 0.5	82.1 ± 14.2
ESP + ITA	25	0.9 ± 0.7	23.9 ± 0.9	0.0 ± 0.0	0.3 ± 0.5	95.4 ± 3.6
GEO	8	0.0 ± 0.0	0.0 ± 0.0	8.0 ± 0.0	0.0 ± 0.0	100.0 ± 0.0
Total	41					93.7 ± 4.2

Model parameters are the mean values obtained with the training sets (N = 166) from 7 iterations: 6 LVs, Q^2^ = 0.648, and RMSEcv = 0.296. For all models, ANOVA *p*-value < 0.05. CHL: Chile; ESP: Spain; GEO: Georgia; ITA: Italy.

## Data Availability

The original data presented in the study are available at CORA. Repositori de Dades de Recerca: Torres Cobos, Berta; Rosell Linares, Mònica; Soler, Albert; Rovira, Mercè; Romero, Agustí; Guardiola, Francesc; Vichi, Stefania; Tres, Alba, 2024, “Stable isotope ratio values (bulk δ^18^O, and δ^13^C and δ^2^H of various fatty acids) of hazelnuts”, https://doi.org/10.34810/data1724.

## Supplementary Materials

The following supporting information can be downloaded at https://www.mdpi.com/article/10.3390/foods13213399/s1. Table S1: Sample information and composition of the seven training (train) and validation (test) sets; Table S2: Mean ± standard deviation and statistical tests (normality test of Shapiro–Wilk, independent samples *t*-test, one-way analysis of variance (ANOVA), U Mann–Whitney and Kruskal–Wallis) with *p*-values to assess the interannual variability of the isotopic ratios for each geographical region; Table S3: Results of the leave 10% out cross-validation of the PLS-DA training models developed on δ^18^O and the δ^2^H, δ^13^C of the main FAMEs data to discriminate samples from three regions of origin. Results are the mean values (± standard deviation) obtained from seven iterations; Table S4: Thresholds of the PLS-DA models (7 training sets) for each class optimized with ROC curves; Table S5: Results of the leave 10% out cross-validation of the PLS-DA training models developed on δ^18^O, and the δ^2^H, δ^13^C of the main FAMEs data to discriminate samples from three regions of origin. Results are the mean values (±standard deviation) obtained from seven iterations; and Table S6: Misclassified and not assigned sample information from each test set.
